# Long-term COVID-19 symptoms and post-vaccination reactions among prolonged COVID-19 patients in the Kurdistan region of Iraq

**DOI:** 10.1016/j.dib.2025.111785

**Published:** 2025-06-17

**Authors:** Aveen Kakamen, Ibrahim Ismael Hamarash

**Affiliations:** a Department of Computer Science and Engineering, School of Science and engineering, University of Kurdistan-Hewler, Iraq; b Department of Electrical Engineering, College of Engineering, Salahaddin University-Erbil, Kurdistan, Iraq

**Keywords:** Long-term COVID 19, KLTCD, Vaccination, Machine learning, Dataset, Kurdistan region

## Abstract

Vaccination has long been recognized as the most effective means for disease prevention, yet concerns have raised globally regarding possible side effects. These concerns were very serious with the COVID-19 vaccines due to the accelerated development process, which did not follow the traditional vaccine lifecycle. This paper describes a dataset that captures a range of long-term symptoms in patients after recovering from the virus including information on vaccination status and self-reported memory issues. The research was carried out in the Kurdistan region of Iraq, and as a result, The Kurdistan Long Term COVID Dataset (KLTCD) was established. The dataset is publicly available online. Basic statistical analysis, measures and machine learning logistic algorithm have been used to evaluate the hypothesis. The established dataset contains numerous features, variables, and labels, making it one of the most information-rich datasets on long-term COVID-19. It may be used to support or refute various hypothesis related to the virus.

Specifications Table

**Table:** 

Subject	Health and Medical Sciences; Epidemiology
Specific subject area	This study examines the relationship between various post COVID-19 symptoms including short-term memory loss and COVID-19 vaccination in individuals recuperating from the virus in Iraq's Kurdistan Region. The study, which used the Kurdistan Long Term COVID Dataset, revealed no link between short-term memory loss and immunization. The results point to the need for more investigation to completely comprehend the long-term consequences of COVID-19 and how it can affect memory loss and other long-term symptoms.
Type of data	Table
Data collection	The Kurdistan Long-Term COVID Dataset (KLTCD) has been established using two data sources. The first source is patient dossiers from Par hospital in Erbil city, the capital of the Kurdistan Region of Iraq. These dossiers include demographic information, past medical history, vaccinations, medical tests, symptoms at the beginning and during COVID-19, and clinical parameters. Thirty-six characteristics were selected with the help of two expert physicians at Par hospital, their efforts have been appreciated in the acknowledgment section of this article. The second source of data was collected through direct communication with patients one year after their recovery. During these calls, we obtained consent for participation and inquired if they had fully recovered or if they still experienced one or any combination of nine symptoms selected by the expert physicians. Both sources were combined to establish the first draft of the dataset. However, since some patients had passed away or many dossiers were incomplete, we applied pre-processing processes. These processes involved eliminating IDs and names to ensure privacy and ethicality and removing null features and records. The remaining data included 3,103 records. The categorical features were then converted to numerical data types to prepare the dataset for analysis using Artificial Intelligence, Machine Learning, and other analysis methods.
Data source location	*University of Kurdistan Hewler, Erbil, Kurdistan, Iraq*
Data accessibility	***Long-Term Follow-Up Data of 3060 COVID-19 Recovered Patients in Kurdistan, Iraq: A One-Year Post-Recovery Study*** Repository name: Mendeley Data Data identification number: https://doi.org/10.17632/ntw6rghz7c.4 Direct URL to data: **Long-Term Follow-Up Data of 3,060 COVID-19 Recovered Patients in Kurdistan, Iraq: A One-Year Post-Recovery Study - Mendeley Data**
Related research article	A.K. Mustafa and I.I. Hamarash, ``Predicting Long-term Covid-19 Symptoms Using Machine Learning: A Case Study in Kurdistan Region of Iraq,'' The Journal of The University of Duhok, vol. 26, no. 2, pp. 605-612, 2023. [Online]. Available: https://doi.org/10.26682/csjuod.2023.26.2.54

## 1 Value of the Data

Recently, predicting outcomes using historical data has been widely achieved through Artificial Intelligence (AI) algorithms [1]. This advancement in AI benefits many aspects of life, including epidemiology. Datasets collected from different parts of the world adds diversity and help reduce bias in AI applications and enable modelling and prediction tailored to individual communities. The KLTCD is valuable for several reasons:

• The data was collected between **March 2020 and December 2023**, providing a longitudinal perspective on post-COVID symptoms.
• The KLTCD is a comprehensive and complete dataset, pre-processed for use with AI and machine learning algorithms. It is designed to build predictive models for the long-term effects of COVID-19, aiding in the prediction of persistent symptoms.
• The dataset provides clinical parameters and long-term symptoms for 3103 patients, which helps in identifying potential biomarkers that can predict long-term consequences of COVID-19. The dataset enables the development of predictive models that can assist healthcare practitioners in identifying individuals at higher risk of experiencing long-term COVID-19 effects. This information is crucial for formulating targeted preventative and treatment strategies.
• The KLTCD contributes to the global body of COVID-19 research by providing a detailed dataset of long-term impact variables within a specific community. This contribution enhances our understanding of Covid-19 pandemic and can influence global health policy and patient care systems.
• While the dataset includes asthma as a pre-existing respiratory condition, future iterations aim to expand this scope to include other conditions such as COPD.

## 2 Background

The COVID-19 pandemic has posed substantial challenges to most communities, and healthcare systems globally. In the beginning, efforts focused on managing the acute impact of the virus, but later evidence revealed that a significant portion of individuals continued to suffer from symptoms even after recovering from the acute phase [2]. Studying this condition requires data collected from individual cases. The National Center for Health Statistics (NCHS) of the United States and the Centers for Disease Control and Prevention published a survey on the prevalence of post-COVID-19 conditions (long COVID) [3]. However, this dataset lacks the features and variables necessary to fully understand the condition, including only age group and symptom duration, making it unsuitable for AI applications. Another survey was conducted in Sri Lanka using mobile phone-based, cross-sectional methods to gather national-level data during the COVID-19 pandemic. The survey focused on public knowledge, behaviour, testing, and vaccine acceptability through automated voice calls in Sinhala, Tamil, and English. However, it primarily included societal-related features and lacked clinical data and information on the long-term effects of the virus [4]. A comparative analysis of global COVID-19 case reports from WHO and JHU dashboards between 2020 and 2022 revealed substantial inconsistencies across 191 countries [5]. While the study offered valuable insights into data reliability and reporting patterns, it primarily dealt with aggregated case numbers. The absence of individual-level clinical details or long-term patient outcomes limits its relevance for studies focused on personalized health impacts or predictive analytics. A study in Assam, India, used machine learning and deep learning on data from 5329 hospitalized COVID-19 patients to identify baseline characteristics linked to severe outcomes and mortality [6]. It addressed the shortage of clinical data during the 2020–2022 lockdown period, aiming to improve patient care and resource prioritization. However, the study focused primarily on acute severity, with limited attention to long-term post-COVID symptoms. There is a severe global shortage of datasets for studying this condition. This motivated us to establish a dataset specifically for this COVID-19 condition.

As AI and machine learning tools are among the most widely used methods to analyse epidemiological data, the KLTCD dataset is prepared and standardized to be suitable for various types of analyses and predictive modelling, including AI and machine learning. This paper specifically focuses on the long-term symptoms experienced by recovered COVID-19 patients and investigating the potential influence of vaccination, including memory-related concerns.

## 3 Data Description

**Fig. 1:**
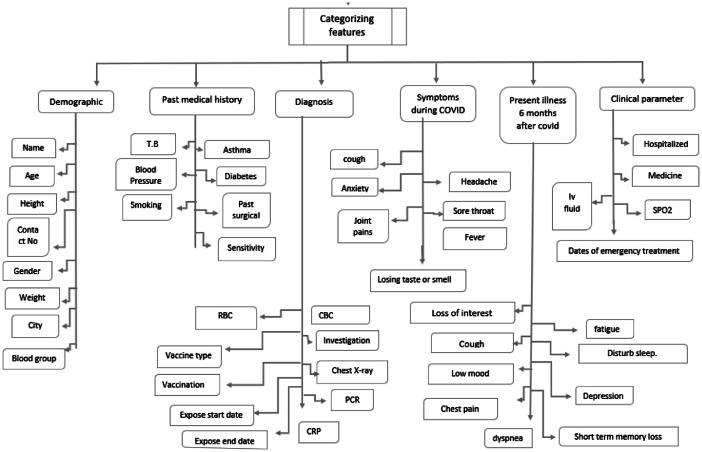
Structural overview of the data collection process.

This dataset includes specific information on 3000 COVID-19 recovery patients from Iraq's Kurdistan Region. The data includes 46 features, 15 of which were rigorously vetted by qualified COVID-19 clinicians and the remaining 31 by committed researchers. The dataset provides a comprehensive picture of the patients' health, recovery progress, and a variety of demographic and clinical characteristics. The use of expert-collected data assures a high level of precision and dependability when analyzing the patients' conditions. Researchers and healthcare professionals can use this comprehensive dataset to gain valuable insights into the recovery patterns of COVID-19 patients in the Kurdistan region, contributing to a better understanding of the virus and enhancing the development of targeted interventions and treatment plans. Below diagram represents data collection process (Fig. 1).

The dataset used in this study includes of 37 features categorized into six groups. Group A (Demographic) includes: name, age, gender (male/female), height, weight, contact number, blood group, and address(city). Group B (Past Medical History) comprises tuberculosis (T.B), blood pressure, diabetes (Type 1/2), sensitivity (allergic to medicine), past surgical history, asthma, and smoking. Group C (Diagnosis) features include red blood cells (RBC), vaccine type, vaccination status (Yes/No), cardiopulmonary resuscitation (CRP), polymerase chain reaction (PCR), chest x-ray, investigation, exposure start date, exposure end date, and complete blood count (CBC). Group D (Symptoms During COVID) includes cough, anxiety, joint pains, fever, sore throat, loss of taste or smell, and headache. Group E (Present Illness Six Months After COVID) includes: shortness of breath (SOB), low mood, fatigue, depression, disturbed sleep, short-term memory loss, dyspnoea, chest pain, and cough. Finally, Group F (Clinical Parameters) includes hospitalization, SpO2 level, IV fluid intake, medication, number of treatment dates, and target.

## 4 Experimental Design, Materials and Methods

The study aimed to investigate the long-term effects of COVID-19 on patient health outcomes, focusing on demographics, medical history, diagnosis, clinical variables, and symptoms during and after COVID [7]. Data were gathered from patient dossiers at Par International Hospital in Kurdistan Region, Iraq. Trained medical personnel collected information directly from patients during hospital visits using standard forms, diagnostic tests and existing hospital records.

Thirty-six variables were selected in consultation with a hospital expert physician, addressing critical research aspects concerning the persistence and severity of symptoms among COVID-19 recovery patients, and identifying factors influencing long-term outcomes. Then, one-on-one patient interviews were conducted to complete the dataset, querying whether any of the suggested nine specified symptoms persisted one year after diagnosis, as recommended by the expert physician.

Throughout both phases of data collection, ethical considerations such as patient confidentiality, informed consent, and data anonymization were strictly followed. To standardize the dataset, preprocessing steps were applied, encompassing cleaning, formatting, and normalization processes to ensure data integrity. Categorical data were converted into numerical formats, followed by refining the dataset to remove null values and normalize data characteristics.

## Limitations

During the second phase of data collection, when contacting patients by phone, a significant number of them were elderly or illiterate, resulting in their family members answering on their behalf or requiring explanations to provide consent.

## Ethics Statement

As the data pertains to health information of individuals, its collection adheres to Salahaddin University-Erbil's ethical regulations outlined in the Operational Guidelines and Procedures of the Human Research Ethics Committee (HREC). The protocol code for this study is 45-266, dated June 24, 2022

## CRediT Author Statement

**Aveen Kakamen:** Methodology, Data curation, Formal analysis, Writing - Original draft, **Professor Ibrahim Hamarash:** Conceptualization, Supervision, Writing - Review & Editing.

## Data Availability

Mendeley DataLong-Term Follow-Up Data of 3060 COVID-19 Recovered Patients in Kurdistan, Iraq: A One-Year Post-Recovery Study (Original data).

## References

[1] Arora A. (2023). The value of standards for health datasets in artificial intelligence-based applications. Nat. Med..

[2] Mustafa A.K., Hamarash I.I. (2023). Predicting long-term Covid-19 symptoms using machine learning: a case study in Kurdistan region of Iraq. J. Univ. Duhok.

[3] National Center for Health Statistics, Centers for Disease Control and Prevention, "Post COVID Conditions." [Online]. Available: https://www.cdc.gov/nchs/covid19/pulse/long-covid.htm [Accessed: 28 March 2024].

[4] Phadnis R., Perera U., Lea V., Davlin S., Lee J., Siesel C., Abeygunathilaka D., Wickramasinghe S.C. (2024). Designing and validating a survey for national-level data during the COVID-19 pandemic in Sri Lanka: cross-sectional mobile phone surveys. JMIR Format. Res..

[5] Liu H., Zong H., Yang Y., Schwebel D.C., Xie B., Ning P., Rao Z., Li L., Hu G. (2025). Consistency of daily number of reported COVID-19 cases in 191 countries from 2020 to 2022: comparative analysis of 2 major data sources. JMIR Public Health Surveill..

[6] Choudhury A., Sarma K.K., Dutta L., Misra D.D., Choudhury A., Sarma R. (2024). Analysis of data of COVID lockdown period: comorbidity and fatality rates in a few districts of Assam, India. Data Br..

[7] Suppakitjanusnt P. (2021). Identifying individuals with recent COVID-19 through voice classification using deep learning. Sci. Rep..

